# Clinical Utility of Positron Emission Tomography Magnetic Resonance Imaging (PET-MRI) in Gastrointestinal Cancers

**DOI:** 10.3390/diagnostics6030035

**Published:** 2016-09-09

**Authors:** Robert Matthews, Minsig Choi

**Affiliations:** 1 Department of Radiology, Stony Brook University Medical Center, Stony Brook, NY 11794, USA; robert.matthews@stonybrookmedicine.edu; 2 Department of Medicine, Stony Brook University Medical Center, Stony Brook, NY 11794, USA

**Keywords:** PET-MRI, positron emission tomography, fluorodeoxyglucose, magnetic resonance imaging, cancer

## Abstract

Anatomic imaging utilizing both CT (computed tomography) and MRI (magnetic resonance imaging) limits the assessment of cancer metastases in lymph nodes and distant organs while functional imaging like PET (positron emission tomography) scan has its limitation in spatial resolution capacity. Hybrid imaging utilizing PET-CT and PET-MRI are novel imaging modalities that are changing the current landscape in cancer diagnosis, staging, and treatment response. MRI has shown to have higher sensitivity in soft tissue, head and neck pathology, and pelvic disease, as well as, detecting small metastases in the liver and bone compared to CT. Combining MRI with PET allows for detection of metastases that may have been missed with current imaging modalities. In this review, we will examine the clinical utility of FDG PET-MRI in the diagnosis and staging of gastrointestinal cancers with focus on esophageal, stomach, colorectal, and pancreatic cancers. We will also explore its role in treatment response and future directions associated with it.

## 1. Introduction

Cancer is a global health issue that affects about 1.7 million patients in United States every year. Current epidemiology suggests that one in two will be affected with this serious life threatening disease once in a life time [1]. Appropriate diagnostic work up and staging is critical in its prognosis and therapeutic success. Computed tomography has been the standard for the diagnosis and staging of cancer for the last three decades. CT scan has a sensitivity of 80%–90% and specificity of 90% to elucidate important abdominal and pulmonary structures [2]. Magnetic resonance imaging (MRI) has improved sensitivity for soft tissue and subtle metastases in brain, liver, and pelvic structures and is used as a problem solving tool in cancer diagnosis and imaging. Over the last decade, novel functional imaging has been incorporated to current anatomic imaging methodology like CT and MRI in cancer diagnosis and treatment.

Positron emission tomography (PET) scans are a noninvasive imaging modality utilizing positron emitting radioisotopes to label molecules and create different images depending on its tissue concentrations. The most common radiotracer used in oncology is F18 fluorodeoxyglucose (FDG) that is a glucose analog with a half-life of 110 min. When these tiny positive electrons encounter a regular negative electron they form a nuclear reaction emitting two photons of high energy at 180°, annihilation rays, that can be detected by the PET crystal detector. FDG is transported via different glucose transporters into the cell then phosphorylated by hexokinase but further glucose metabolism is prevented by the fluorine atom and FDG is trapped inside the cell. Metabolically active cancer cells use more glucose and express higher levels of glucose transporter (GLUT-1) than normal cells, hence, more FDG is expressed in malignancies [3]. In the background of normal cellular uptake, these foci of malignancy appear as hypermetabolic areas on the PET scanner reflecting areas of higher glucose metabolism. Each tissue in the body has its own background glucose use with the brain being the most intense, and the kidneys actively excreting it through the urinary system. The PET scan is obtained during fasting conditions to avoid intense glucose uptake driven into the skeletal muscles [4]. The amount of radiotracer within a given lesion can be quantified to assess clinical response and aid in determining the degree of malignancy using the measurement of standardized uptake value (SUV) [5].

PET imaging alone has several limitations. There is low spatial resolution and difficulty interpreting abnormalities in the setting of physiological FDG uptake in normal anatomical structures, as well as normal variants. In addition, not only does malignancy show abnormal uptake, but other pathological processes including inflammation and infection can also have prominent FDG uptake [6]. Also the degree of malignancy correlates with the intensity of FDG uptake. Highly aggressive tumors have intense uptake while less aggressive tumors such as prostate cancer or low grade lymphoma have only mild uptake. In addition, many benign tumors, most notably thyroid adenomas and benign primary parotid tumors, may have intense focal uptake [7]. To compensate for all these limitations, CT was added to the PET scan to provide both higher sensitivity as well as higher specificity. PET-CT is currently used for initial staging of cancer, evaluating the response to chemotherapy and radiation therapy, provide prognosis, and surveillance of recurrent disease. Since functional imaging can provide much faster response than anatomic changes to targeted therapies, PET has been incorporated in development of new cancer drug discovery and has been used in early clinical trial development [8]. However, use of PET-CT has a relatively high radiation exposure level that is often 2–3 times the administered FDG radiation levels depending on the CT protocol used and may lack anatomic details in soft tissue of head and neck and pelvic structures. A low dose CT protocol would have a much lower radiation exposure compared to a fully diagnostic CT protocol [9].

## 2. PET-MRI Scanner

MRI uses magnetic fields with radiofrequency pulses to produce computer images based on stimulating water molecules in the body. MRI is considered the best imaging modality for soft tissue pathology. It is particularly useful in assessing abnormalities in pelvic organs such as the prostate gland and uterus, abdominal organs such as liver, biliary tract, kidneys, spleen, and pancreas. MRI is excellent at musculoskeletal abnormalities making it the standard in evaluating for ligament tears, occult fractures, myopathy, and various forms of arthritis. These strengths in MRI make it ideal for evaluating prostate cancer, gynecological cancers, hepatocellular cancer and liver metastases, renal cell carcinoma, pancreatic adenocarcinoma, soft tissue sarcomas, certain primary bone cancers, multiple myeloma, malignant melanoma, and other malignancies [10].

The introduction of the PET-MRI scanner provided an important diagnostic imaging modality combining the metabolic parameters of PET scanning with the superior soft tissue imaging quality of MR imaging. The first PET-MRI scanners consisted of two separate imaging modalities united by a common gantry or bed so that the patient would not move between the studies. Only one camera was operating at a time, significantly lengthening the study and allowing few patients to be imaged during the day. With the launch of the Biograph mMR, Siemens Imaging allowed a fully integrated hybrid imaging that acquired both the PET and the MRI images simultaneously. Besides reducing the acquisition time in half, the hybrid images lessened misregistration artifact that occurred while moving the patient. This was also an advantage over PET-CT where the CT images were obtained first, followed by PET acquisition that could lead to changes in normal abdominal organs due to bowel movement and bladder filling [11]. Another substantial change was the significant reduction in ionizing radiation since only the PET portion of the study added to exposure without the high energy of the CT scanner. Radiation is reduced by 40%–60% which is important in patients that require multiple scans, as well as, pediatric patients who have a longer life to accumulate radiation exposure. However, new complexities emerged since a variety of MRI sequences had to be chosen for imaging. The patient was added with new discomfort with multiple MRI coils placed on the body and some sequences requiring breath holds.

Since PET-MRI is an emerging technology, no large prospective studies validating FDG PET-MRI in oncologic patients are currently available. Initial pilot studies were intended to verify that PET-MRI imaging was equivalent to PET-CT imaging in lesion detection and feasible in clinical situations. These studies were done to justify the substitution of PET-MRI into cancer assessment instead of PET-CT scanning. However, as familiarity with the PET-MRI camera developed, new sequences and new techniques were introduced giving PET-MRI an advantage over PET-CT in many malignancies [12,13,14,15]. Universal acceptance of PET-MRI imaging will require not only large prospective trials, but also acceptance by the individual clinician. PET-MRI is a new modality and both radiologists and clinicians are still learning its value and limitations. Most oncologists and cancer specialized physicians were trained and have experience understanding the complex language of PET-CT, but now feel uncomfortable in ordering, reviewing, and grasping PET-MRI imaging. For the imaging center, the cost of PET-MRI is significantly higher in terms of machinery, maintenance, and operation. In this review, we will focus on emerging data in diagnosis and treatment of using PET-MRI in gastrointestinal cancers.

## 3. PET-MRI Imaging Protocols

Imaging protocols for PET MRI varies in different institutions and standards are controversial. All PET-MRI scanners require a fast view localizer followed by a Dixon AC sequence for PET attenuation correction. We used a Siemens mMR hybrid PET-MRI scanner with 3T magnet. A typical base of skull to mid thigh body protocol requires five separate bed stations or acquisition stations where all MRI sequences must be the same for each bed using body coils. Typically, T1 vibe and T2 haste are the mainstay of acquisition with or without fat suppression [16]. At our institution we use a T1 radial vibe with fat suppression in a transaxial orientation which does not require breath holds and is sensitive in detecting lung nodules. We follow this with T2 haste without fat suppression in a transaxial orientation. To aid the T2 transaxial images we also acquire a T2 turbo spin echo sequence (TSE) in the coronal plane. Many institutions use diffusion weighted images on the whole body sequence which we abandoned since this sequence did not offer any additional information that the PET portion did not provide. For spinal lesion imaging, we obtain a short tau inversion recovery (STIR) sequence in a sagittal orientation limited to the vertebrae which is excellent at depicting malignancy. Some institutions obtain entire body STIR imaging. Additional sequences may also be obtained depending on clinical scenario. Our entire PET-MRI body protocol is 5 min in length per bed position which totals approximately 25 min of imaging time. We also have chosen to include the entire brain. In addition at our institution, we add special dedicated neck, liver, and pelvic MRI sequences with IV contrast that last 30–40 min of additional time when imaging head and neck, liver, rectal, and gynecological malignancies.

## 4. Esophageal Cancer

After initial diagnosis of esophageal cancer from upper endoscopy with biopsy, the current methods of staging this malignancy involves CT chest and abdomen, endoscopic ultrasonography (EUS), and PET-CT with FDG. EUS is essentially used to determine primary tumor depth and involvement by identifying layers of the esophageal wall. Clinical distinction for T1 and T2-4 diseases is important since neoadjuvant chemoradiotherapy is used in most locally advanced esophageal cancer followed by surgical resection. FDG PET-CT offers an alternative test because it may detect small lymph nodes not detected by size criteria but are metabolically active, but overall, PET-CT and CT are limited in detecting local lymph nodes as well as peritoneal spread of disease. PET-CT is superior at detecting distant metastases in the liver and lungs compared to CT scan alone [17].

**Figure 1:**
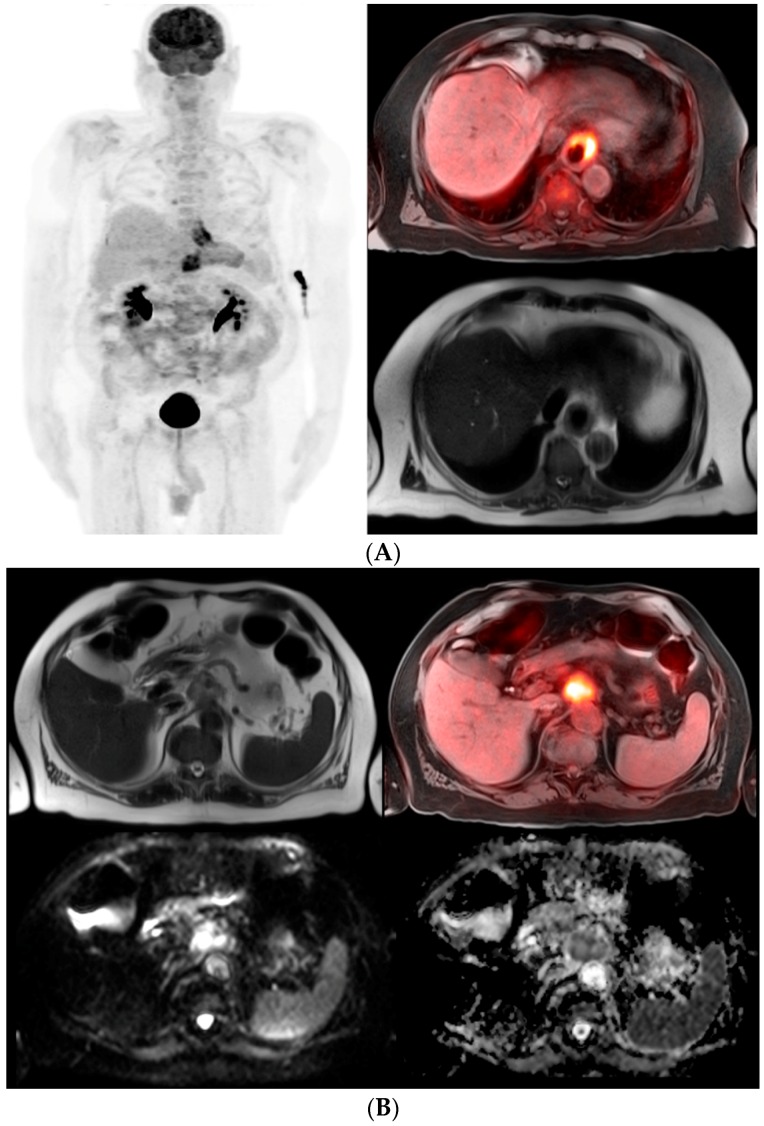
(**A**) 71-year-old male with a history of esophageal adenocarcinoma with an esophageal stent. PET maximum intensity projection (MIP) images demonstrate two abnormal FDG foci corresponding to the distal esophageal mass and an upper abdominal metastatic lesion (*left image*). The fusion PET-MRI transaxial image using T1 with fat suppression shows prominent abnormal FDG uptake in the distal esophageal cancer (*top right*). The T2 haste transaxial image demonstrates eccentric esophageal wall thickening (*bottom right*); (**B**) Metastatic lymph node from esophageal adenocarcinoma. The T2 haste transaxial image shows an enlarged lymph node located superior to the celiac axis of the aorta (*top left*). Fusion PET-MRI image using T1 with fat suppression reveals prominent abnormal FDG activity consistent with metastasis (*top right*). Diffusion weighted images demonstrate hyperintense signal in the enlarged lymph node (*bottom left*) with corresponding low signal on the apparent diffusion coefficient map (*bottom right*) which is consistent with malignancy.

The rapid evolving PET-MRI imaging modality with its superior soft tissue resolution and metabolic activity has potential in initial staging of esophageal cancer. With the improvement of MRI cameras, protocols, and techniques that resulted in high quality imaging, staging of esophageal cancer has significantly improved. Diffusion weighted imaging with the conjunction of high resolution T2 weighted MRI images, the extent of tumor wall invasion as well as local and distant metastases have improved. Other sequences like STIR and TSE have also improved diagnostic accuracy. MRI contrast agents may even detect small lesions which are difficult to see [17,18]. Various MRI imaging protocols for esophageal cancer have been established. In one small series of 19 patients, PET/MRI imaging demonstrated comparable accuracy for T staging (80%–86%) with EUS and higher accuracy for N staging (83% sensitivity) compared to both EUS and PET-CT (75%–67%). In addition, PET-MRI was able to detect distant metastatic disease as shown in Figure 1 [19].

Use of MRI in treatment response in esophageal cancer is currently evolving. Ayoyagi et al. have shown that high ADC values were associated with better response to chemoradiotherapy in esophageal cancer patients and higher survival rates [20]. This area is definitely a critical area in oncology since selecting patient with complete pathologic response who may not require a morbid surgical resection with high complication rate can improve patient outcome. Future studies on role of both functional MRI and PET may clarify the potential clinical implication in treatment assessment in esophageal cancer.

## 5. Stomach Cancer

The preoperative staging of stomach cancer has been traditionally performed with endoscopic ultrasonography and contrast enhanced CT. EUS has been valuable in assessing tumor wall invasion and in the detection of lymph node involvement. EUS has the ability to evaluate the tumor in close proximity viewing all five layers of the wall with the ability of performing biopsy for lymph node involvement. One weakness of EUS is lack of metastatic assessment in more advanced cancer [21].

**Figure 2:**
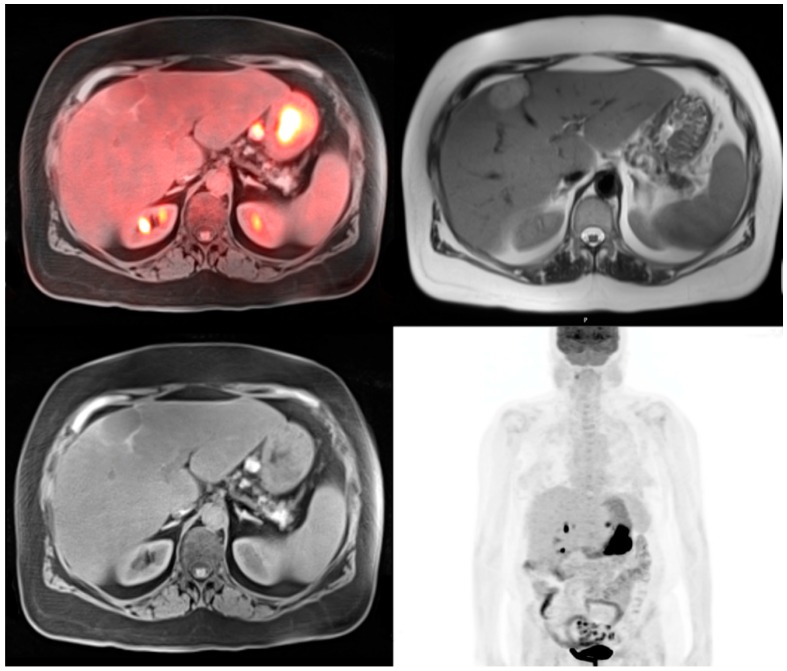
64-year-old female for initial staging of gastric cancer. The PET-MRI transaxial fusion image shows intense hypermetabolic activity in the gastric cancer with SUV 29.2 and a hypermetabolic enlarged gastrohepatic lymph node (*top left*). T1 radial vibe transaxial image with fat suppression shows the gastric cancer to be of low to isointense signal and the metastatic lesion to be hyperintense (*bottom left*). T2 haste transaxial image shows a hyperintense liver lesion that was not active on the PET images which is consistent with a hemangioma (*top right*). The PET MIP image shows the intense activity in the stomach with activity in the adjacent gastrohepatic lymph node (*bottom right*).

CT has the ability to assess for locoregional involvement, as well as, distant metastases. It has limitations in T staging but can provide anatomic information of metastatic lymph nodes based on size and morphology criteria. The addition of PET imaging can detect normal size lymph nodes with cancer involvement. PET is more suitable in detecting distant metastatic disease, especially hepatic metastases [22,23]. In recent literature, MRI has shown good diagnostic performance in both tumor and lymph node staging with better results with newer sequences [24]. PET-MRI imaging using newer MRI sequences has a potential for better tumor, nodal, and distant metastatic involvement. Early MRI imaging of the stomach was difficult due to respiratory motion and bowel peristalsis. With new free breathing sequences, this problem has been mostly eliminated. Huang et al. did a meta-analysis of 439 patients for using MRI in T and N staging for gastric cancer. Pooled analysis showed the sensitivity of MRI for distinguishing T1-2 vs. T3-4 was 93% with specificity of 91% however for N staging sensitivity was 86% with specificity of only 67% [24]. Figure 2 illustrates a case of gastric cancer using PET-MRI as a staging modality. Future studies are needed to find out if PET-MRI can improve the nodal staging specificity in this disease.

Localized gastric cancer is treated with either pre-operative systemic chemotherapy followed by surgical resection or surgical resection followed by adjuvant chemotherapy. Since there is a very small percentage who will have complete pathologic response to systemic chemotherapy alone, there is less enthusiasm in assessing treatment response in gastric cancer. Data is emerging on the use of PET-MRI in the treatment response to chemotherapy and pilot study of 11 patients showed a synergetic advantage of FDG PET-MRI in advanced gastric cancer with good correlation to chemotherapy response [25,26,27].

## 6. Colorectal Cancer

The initial evaluation of colon cancer often involves colonoscopy with biopsy and contrast enhanced CT for assessment of liver lesions. Contrast enhanced CT has an acceptable detection of liver metastases but may miss detection in up to 7% of the patients and may overestimate liver involvement of others. Extrahepatic abdominal metastases are commonly missed by CT and has difficulty in differentiating post-surgical changes from recurrence. PET-CT has been shown to be slightly more sensitive than CT in liver metastases and better at detecting extrahepatic abdominal metastases. However, due to the variable uptake in hepatic lesions, PET-CT may miss small lesions [28].

**Figure 3:**
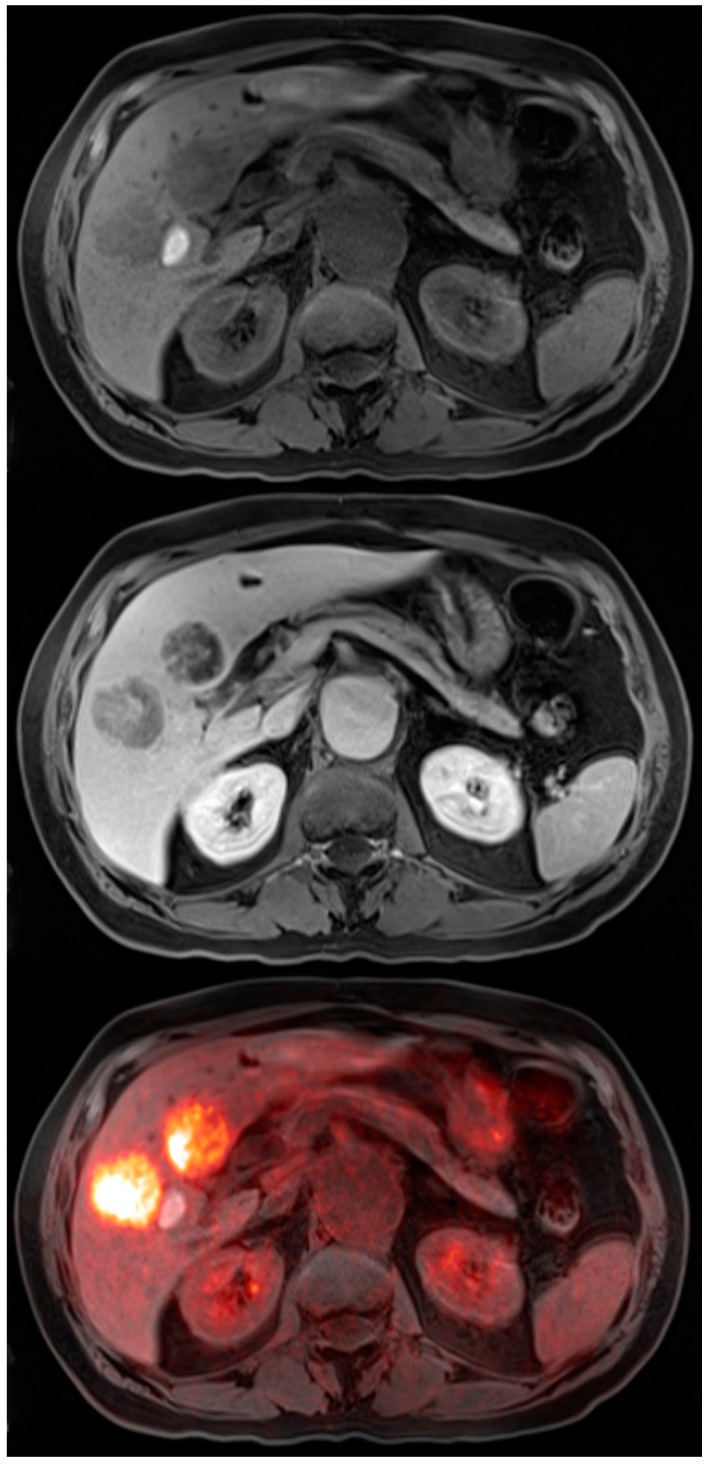
72-year-old male with colon cancer. T1 precontrast transaxial image shows two low signal masses in segment 4B and 5 of the liver (*top*). T1 post contrast transaxial imaging with gadoxetate disodium reveals heterogenous enhancement of these two masses consistent with metastatic colon cancer (*middle*). PET-MRI transaxial fusion image confirms malignancy by demonstrating intense FDG activity (*bottom*). Also note the adjacent benign hemorrhagic cyst with high T1 signal that does not exhibit increased metabolic activity on the fusion images.

Unenhanced standard MRI sequences including T1 and T2 weighted images can accurately characterize and detect most focal liver lesions and can differentiate between solid and non-solid lesions as shown in Figure 3. Colorectal liver metastases are usually hypovascular, and therefore do not demonstrate significant enhancement with gadolinium based contrast agents [29]. MRI is more advantageous in the assessment of liver metastases when functional imaging like DWI and appropriate hepatobiliary contrast agents are used. Liver specific MRI contrast agents have the highest detection rate for focal lesions if the patient is being evaluated for curative surgical resection [30]. Kang et al. showed that PET-MRI added value to CT scan in 51 patients with colorectal cancer and led to 21% of patient changing therapeutic plans.

**Figure 4:**
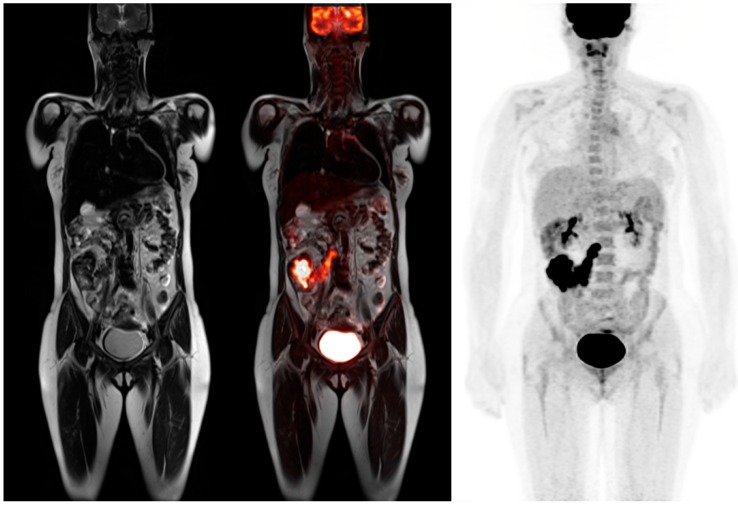
45-year-old female for initial staging of colon cancer. T2 coronal image shows a large right colon mass with multiple low signal intensity lymph nodes (*left*). PET-MRI coronal fusion images better reveal the colon mass and chain of mesenteric lymph nodes that extends to the paracaval region (*middle*). PET MIP image shows the prominent FDG activity in the colon cancer and lymph node metastases (*right*).

The risk of lymph node involvement increases with depth of tumor wall invasion ranging from 15% to 65%. Traditional mesenteric lymph node metastases are based on nodal size and shape with 5 mm being the optimal size. Both CT and MRI have detection capabilities for nodal metastases. However, reactive lymph nodes are common and can confuse interpretation of metastatic spread [31]. FDG PET-MRI has been shown to be useful in characterizing indeterminate lesions including borderline enlarged lymph nodes and small hepatic metastases. The absence or presence of FDG uptake in mesenteric lymph nodes leads to a more accurate assessment of local metastases as shown in Figure 4. In addition, PET-MRI can provide more detailed T staging than PET-CT alone with better characteristics of colorectal wall invasion [32,33].

FDG PET-MRI may have a special role in staging and restaging of rectal cancer. At our institution, in addition to full body PET-MRI scanning, we obtain dedicated pelvic MRI sequences with intravenous contrast after the body images on all rectal cancer patients. During the dedicated pelvic MRI sequences, we continuously acquire the PET signal which leads to a high resolution image of the pelvis. This leads to a high sensitivity in detecting small pelvic lymph nodes. MRI does not have a high degree of accuracy in distinguishing T1 from T2 tumors, but T3 tumors that extend beyond the muscularis propria can be easily detected. In addition, MRI correctly characterizes T4 tumors that spread into visceral peritoneum, adjacent organs, or the levator musculature. MRI has limited sensitivity and specificity in determining pelvic lymph node spread based on changes in size, morphology, or enhancement. The addition of PET imaging showing abnormal metabolic activity in regional lymph nodes adds to a more accurate assessment [34]. These are very important clinical parameters in treatment decision where neoadjuvant chemoradiotherapy is offered in rectal cancer patients with T3 or higher or lymph node metastasis while T1-2 patients undergo surgical resections. The functional images are also useful in the use of radiation therapy and dose intensification during neoadjuvant treatments.

## 7. Pancreatic Cancer

**Figure 5:**
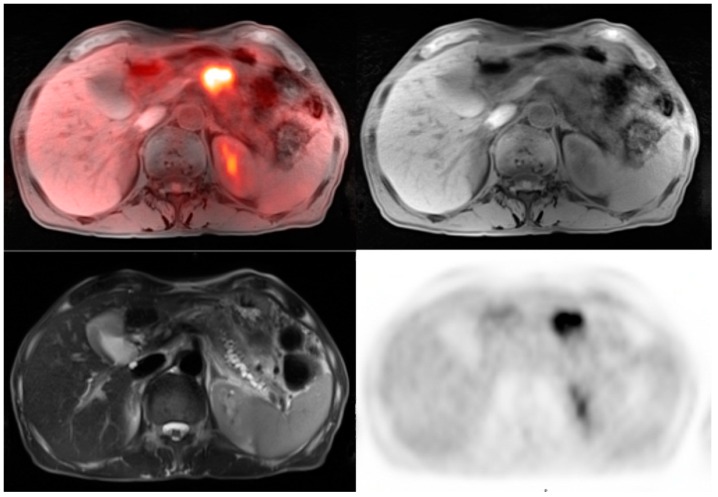
62-year-old male for initial staging of pancreatic adenocarcinoma. The PET-MRI axial fusion image shows a large hypermetabolic mass in the body of the pancreas (*top left*). The T1 axial image demonstrates a mass in the pancreatic body that is isointense to the normal pancreatic tissue (*top right*). The T2 axial images show considerable main pancreatic duct dilatation (*bottom left*). The PET axial image displays intense metabolic activity within the pancreatic tumor relative to the liver activity with standardized uptake value of 8.7.

Pancreatic cancer is diagnosed using multiple modalities including abdominal ultrasound, endoscopic ultrasound, endoscopic retrograde cholangiopancreatography, CT, MRI, and PET/CT. For staging purposes, current recommendations include cross-sectional imaging such as contrast enhanced CT or MRI. PET-CT imaging has played an important role in characterizing pancreatic lesions and its metastatic spread. Most malignant tumors of the pancreas are active on FDG PET imaging and PET-CT has much higher sensitivity and specificity than contrast enhanced CT [35] as illustrated in Figure 5. MRI is frequently employed to evaluate pancreatic lesions because of its superior soft tissue contrast resolution. MRI can additionally evaluate for pancreatic duct dilatation and collateral vein dilatation in addition to assessing necrosis, cystic changes, and fibrosis [36].

Nagamachi et al. examined 119 patients with pancreatic lesions and compared PET-MRI to PET-CT images. PET-MRI demonstrated higher accuracy of 96% vs. 86% in the assessment of pancreatic tumors in comparison to PET-CT [37]. PET-MRI offers better lesion detection, characterization, and definition. Even non-contrast enhanced PET-MRI body sequences offered superior image quality compared to PET-CT [38]. FDG PET-MRI could accurately detect encasement of vessels, infiltration of surrounding structures, and invasion of the common bile duct that are not well delineated on PET-CT [37]. It seems like both PET-CT and PET-MRI are cost effective by determining patient who undergoes futile laparotomy by detecting distant metastases [39].

## 8. Conclusions

With the recent introduction of an integrated PET-MRI, this novel imaging modality is available for the detection, staging, and evaluation of gastrointestinal cancers. PET-MRI has a significant reduction in radiation exposure compared to PET-CT which may benefit younger patients or patients that require frequent repeat PET imaging. MRI offers superior soft tissue resolution compared to CT in rectal and pancreatic cancer. As novel hybrid imaging technology improves, PET-MRI has great potential in the assessment of esophageal cancer, colorectal cancer, stomach cancer, and pancreatic cancer in terms of primary lesion evaluation, nodal involvement, and liver metastases with a high degree of diagnostic accuracy and specificity. Larger clinical validation studies are needed to make PET-MRI an integral part of gastrointestinal cancer patient management in the near future.
